# Using SMILES strings for the description of chemical connectivity in the Crystallography Open Database

**DOI:** 10.1186/s13321-018-0279-6

**Published:** 2018-05-18

**Authors:** Miguel Quirós, Saulius Gražulis, Saulė Girdzijauskaitė, Andrius Merkys, Antanas Vaitkus

**Affiliations:** 1 Departamento de Química Inorgánica, Universidad de Granada, 18071 Granada, Spain; 2 Institute of Biotechnology, Vilnius University, Saulėtekio al. 7, 10257 Vilnius, Lithuania; 3 Faculty of Mathematics and Informatics, Vilnius University, Naugarduko st. 24, 03225 Vilnius, Lithuania

**Keywords:** Crystallography Open Database, Open access to scientific data, Crystal structure database, Molecular structure, SMILES, Substructure search

## Abstract

Computer descriptions of chemical molecular connectivity are necessary for searching chemical databases and for predicting chemical properties from molecular structure. In this article, the ongoing work to describe the chemical connectivity of entries contained in the Crystallography Open Database (COD) in SMILES format is reported. This collection of SMILES is publicly available for chemical (substructure) search or for any other purpose on an open-access basis, as is the COD itself. The conventions that have been followed for the representation of compounds that do not fit into the valence bond theory are outlined for the most frequently found cases. The procedure for getting the SMILES out of the CIF files starts with checking whether the atoms in the asymmetric unit are a chemically acceptable image of the compound. When they are not (molecule in a symmetry element, disorder, polymeric species,etc.), the previously published cif_molecule program is used to get such image in many cases. The program package *Open Babel* is then applied to get SMILES strings from the CIF files (either those directly taken from the COD or those produced by cif_molecule when applicable). The results are then checked and/or fixed by a human editor, in a computer-aided task that at present still consumes a great deal of human time. Even if the procedure still needs to be improved to make it more automatic (and hence faster), it has already yielded more than 160,000 curated chemical structures and the purpose of this article is to announce the existence of this work to the chemical community as well as to spread the use of its results.

**Electronic supplementary material:**

The online version of this article (10.1186/s13321-018-0279-6) contains supplementary material, which is available to authorized users.

## Background

The determination of atomic positions in crystalline materials using diffraction methods is one of the most widely used tools to reveal the internal structure of matter. As a result, the scientific community has generated a huge amount of structural data since the first diffraction experiments at the beginning of the 20th century. The importance of organising this vast set of data to make it easier to find any particular piece of information was recognised several decades ago, before the Internet era, when the Cambridge Structural Database (CSD) [1, 2], the Inorganic Chemistry Structural Database (ICSD) [3] and CRYSMET [4] were created. These databases have been historically devoted, respectively, to organic and metal–organic compounds, purely inorganic compounds and metals and alloys, although a partnership between the CSD and the ICSD has been recently announced [5]. They are developed following a closed model, a paid subscription being necessary to use them for data mining. Individual files may be downloaded from these databases at no charge (provided that the information about the existence of the file has been somehow previously found), but such data cannot be further distributed in any way. In contrast, the Protein Data Bank (PDB) [6] which collects biopolymer structures follows an open model and is freely accessible through a Web interface.

The importance of making scientific data and scientific knowledge open to everybody and free of most licensing and copyright barriers is being recognised by a growing number of people and institutions. In this sense, the United Nations Educational, Scientific and Cultural Organisation (UNESCO) has a commitment for the promotion and support of open access to scientific information [7]. The application of this principle to crystallographic data was the reason for the birth of the Crystallography Open Database (COD) [8, 9], a collection of Crystallographic Information Format (CIF) [10] files (around 390,000 at the moment of writing this article) offered to the scientific community on its main website [11] and several mirrors, using an open access distribution model. The goal of the project is to collect all experimentally determined crystal structures of the so-called “small molecule” compounds, thus excluding only the macromolecular biological compounds already accessible through the PDB, making the data freely available to anyone and also breaking the rather artificial separation between organic, metal–organic, inorganic and metallic.

Data in the COD are organised as a *Subversion* [12] repository of CIF files backed by a relational MySQL database [13]. The database stores some of the information extracted from the CIF files such as unit cell parameters, chemical composition or bibliography and, in turn, offers one the ability to reference these values in search queries. The most simple searches may be carried out using the above mentioned Web interface and the more complex ones may be performed by directly querying the database through the powerful MySQL command line by addressing the query to the sql.crystallography.net server as the passwordless cod_reader user [14].

While the search methods outlined above might be sufficient for crystallographers, many of the potential users of the COD are chemists and, as a result, they are more interested in the chemical features of the crystallised compounds than in the purely crystallographic facts. For organic and metal–organic chemists, the chemical features of the compound are mostly defined by the statement of how atoms are directly bounded to each other: this is the so-called “chemical connectivity” or “molecular structure”. Hence, a chemist is more likely to be interested in the particular association of atoms (functional groups, coordination environments) than in space groups or unit cell parameters. Looking for entries in the database containing one of such atom associations is called a substructure search, something that requires extraction of additional information from crystal structures to enable efficient search in relational databases. The establishment of the chemical connectivity of database entries is not only useful for performing substructure searches, but can be used for similarity searches (a precise definition of this may be found in reference [15]) and also for chemically identifying the present species, which allows the cross-linking of the COD with other chemical, non-crystallographic, databases: in this way, a number of the COD entries for which the chemical identity had been established was cross-linked with the corresponding entries in the ChemSpider database [16].

Therefore, in order to perform a substructure, similarity or full chemical structure search in the database, it is necessary to first establish the chemical connectivity of the entries and store it in an appropriate format. CIF format specification defines methods of describing both the connectivity and the geometry of chemical moieties by the means of the _chemical_conn_bond_* data items [17]. However, these items are not usually automatically created by the software used for refining crystal structures and the authors of the CIFs virtually never bother to add them to the files (only three such entries are in the COD, as of revision 199925). Hence, it is necessary to resort to derive the connectivity from atom coordinates with the possible aid of the _geom_bond_* data items (present in $\sim$ 85% COD CIFs), which in most cases are just a list of automatically calculated distances for pairs of atoms closer than the sum of atomic radii plus some offset and without any bond order information. The list may have been manipulated by the CIF authors either to express their idea of chemical bonding or to calculate non-bonding distances that appear in this case in an inadequate place.

On the other hand, the concept of chemical connectivity is not absolutely and unambiguously based on the objective features of a given chemical species, but includes a lot of conventions about which kind of interactions are considered chemical bonds and which are not. The concept is more easily defined when we work inside the boundaries of the valence bond theory (VBT) in which chemical bonds are established between pairs of atoms with a definite bond order (single, double, triple) for each bond. This theory finds its best application in the field of organic chemistry, thus there exists a virtually universally accepted way for representing the chemical connectivity of organic compounds.

We must note nevertheless that there are many chemical species for which the VBT does not give an adequate description. In purely ionic or metallic compounds, bonds are not established between pairs of atoms, but each atom interacts in a similar way with all other atoms in the neighbourhood. There are also cases in which there is no clear and universally accepted criterion for deciding if the interaction between a pair of atoms must be considered a chemical bond (metal–metal bonds, some organometallic species, ...). We may also find compounds such as boranes or metallocenes with polycentric bonds as well as cases where the bond order value is something open to discussion, for example, metal carbonyls or compounds with electronic delocalisation that do not fit in the aromatic compound category. Because of that, we need to establish some criteria about how we are going to represent the chemical connectivity of all these troublesome species or, at least, for the compound families more frequently appearing in the database, this being one of the main subjects of the present article. A second part will be devoted to the progress made so far in extracting the chemical connectivity from the COD entries, a process that nowadays still requires a great deal of human intervention, even if computer-aided.

We should mention that the problem outlined in this manuscript has also been faced by the above mentioned closed-access databases, in particular by CSD (in principle, ICSD and CRYSTMET have no need to deal with chemical connectivities) and they have developed an elaborated algorithm to deduce chemical structures from crystal data [18], certainly more elaborated than what is described here, but even in this case the authors report a degree of success that is not high enough to avoid the participation of human editors. The article does not mention anything about the possibility of obtaining the software that implements the described algorithm.

A molecular representation has already been created for a significant fraction of the COD (above 160,000 entries at the moment of writing this article), this COD subset hence being available for substructure search. We think it is important to communicate to the scientific community the existence of this ongoing work at this stage and to share the results obtained so far. The open nature of the COD makes it possible for every reader to collaborate on this work, contributing with his/her own pieces of software or even designing alternative procedures from scratch.

## Methods

### Format and conventions

 For representing the chemical connectivity of the chemical species contained in the COD, we have chosen the Simplified Molecular Input Line Entry Specification (SMILES), a very widely used format to store this kind of information that has an open specification [19], which is virtually identical to the original specification created by Daylight Chemical Information Systems [20]. This format has further advantages: it is relatively compact and it is readable and editable both by computers and human beings, which makes it especially suitable for being created and/or curated by advanced computer programs, simple scripts or manual editing, a combination of all being currently used for the COD entries. We acknowledge, of course, that the SMILES format has its drawbacks: it is based on the VBT and hence, it is troublesome to represent chemical species that do not fit in this theory, but, as stated in the introduction, the concept of “chemical connectivity” itself is tightly linked to the VBT and we will probably find this drawback in any other existing alternative format that uses the same theory. Also, SMILES cannot properly represent polymeric or extended species. Some extensions have been proposed to overcome this limitation, like the use of the “&” character mentioned in section 6.2 of the OpenSMILES specifications [19] or the annotations defined in the CurlySMILES language [21], but such extensions are not part of the official specification and are unlikely to be recognised by many cheminformatics tools (that may regard them just as syntax errors). Moreover, even with the use of these extensions it is still probably impossible to cover the enormous variety of topological connectivities that may occur in polymeric compounds.

Another widely used format for the representation of chemical connectivities is the IUPAC International Chemical Identifier (InChI) [22], that is intensively developed nowadays involving projects for better representation of inorganic compounds and polymers [23]. However, we have chosen SMILES over InChI as the latter is not well suited for easy human inspection and manipulation.

The number of reasons one could find to regard the SMILES representation of a given chemical species as being difficult, unclear or not unique is virtually infinite, hence trying to design some universal rules that can apply to any conceivable situation is probably an impossible task. Instead of it, we have decided to use a more practical ad hoc approach, establishing conventional representations for the most frequently found cases. In such representations, we have tried to reproduce, as much as possible, the image that we think most chemists will have in their minds for a particular chemical species, giving priority to such representation over others, even over those that might be more suitable for computer manipulation. We are aware that our choices for the representation of some kinds of chemical moieties are not unique and other acceptable choices may exist in several cases. But probably the most important thing is that analogous criteria are applied to analogous compounds so chemical connectivity is assigned in an uniform way. In the following paragraphs we describe the SMILES conventions that we are using to represent compounds belonging to some of the most populous categories found in the COD.

#### Aromatic organic compounds

**Fig. 1:**
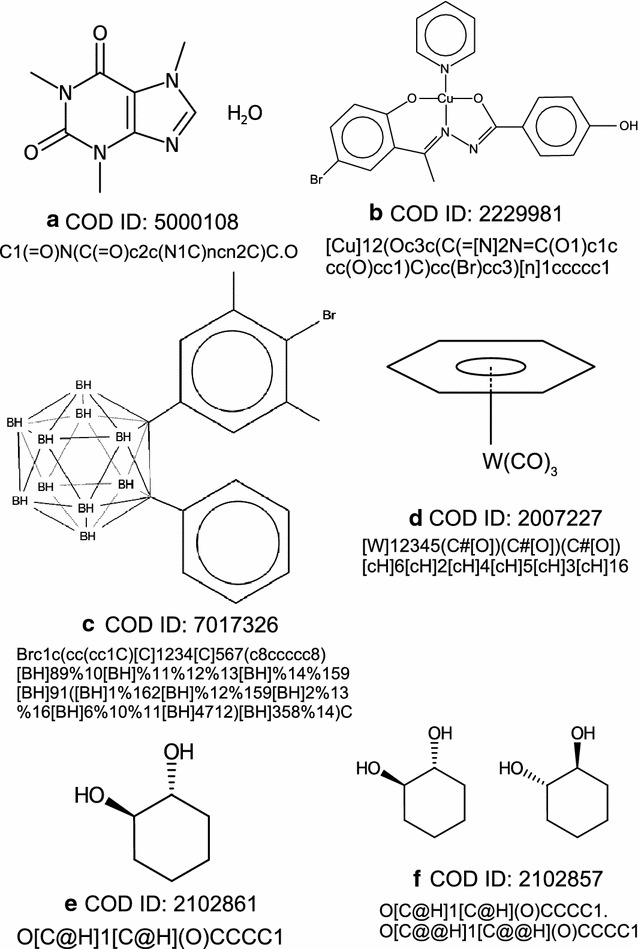
Structural formulae and SMILES strings of some compounds from the COD. **a** Caffeine as an example of compound with arguable aromaticity. **b** A coordination compound including neutral and anionic donor atoms. **c** An example of borane cage compound. **d** An organometallic with η-6 and carbonyl ligands. **e** A pure enantiomer of a chiral compound. f) Both enantiomers of a compound present in a racemic crystal

Organic compounds are usually well explained by the VBT and hence, their SMILES representation seldom poses serious problems. Aromaticity, even if it implies the existence of delocalised bonds, is included in the SMILES specification by using lower-case characters for representing atoms in aromatic rings, even if the alternative choice with explicit alternating single and double bonds (Kekulé-like) is also acceptable and used for example in PubChem [24]. To consider a ring as aromatic, we have established as a necessary condition that the number of involved $\pi$-electrons must obey the Hückel’s rule, hence species such as cyclobutadiene or cyclooctatetraene are not regarded as aromatic. Besides this, these $\pi$-electrons are required to be effectively delocalised, what may be interpreted as the existence of alternating single and double bonds. But there are cases in which the formal alternation between single and double bonds is broken, typically by the presence of a formally single bonded endocyclic heteroatom and/or an exocyclic double bond, which implies that the aromatic character of the ring is open for discussion. For situations like these, the SMILES specification considers both choices (aromatic or non-aromatic) as acceptable. In general, we have considered that an endocyclic singly bonded N, O or S atom does not break aromaticity, while compounds with exocyclic double bonds are considered aromatic if at least one single bond comprised between two double bonds remains in the ring, non-aromatic representation having been chosen otherwise: see Table 1 and Fig. 1a for some representative examples. When the aromatic/non-aromatic dilemma arises for a single atom with single bonds towards two aromatic rings, that atom is regarded as non-aromatic. The idea is always: how would the majority of chemists draw this compound with a chalk on a blackboard? Fullerenes are regarded as a particular case of aromatic compounds, but the presence of atoms linked to the carbon framework breaks aromaticity for the affected carbon atoms and possibly also for some neighbour ones, therefore we have to decide about aromaticity designators for these compounds on a case-by-case basis.

#### Coordination compounds

When a formally non-charged atom links to a metal, it uses an electron pair that is not shared in the free ligand and, as a consequence, this atom forms one extra bond above its standard valence, as defined in the SMILES specification, and therefore square brackets should be used for it. On the other hand, if the binding atom is formally anionic (formal substitution of an H atom by a metal atom), we usually find the number of bonds equal to the expected valence and so there is no need to use square brackets if the donor atom belongs to the organic subset. The subject becomes somewhat more complex when the same atom simultaneously binds to two or more metal atoms. Nevertheless, the rule to conform to the SMILES specification remains the same: square brackets are used if the total sum of bond orders is not equal to the standard valence. This rule is also applied if the binding atom belongs to an aromatic ring: we use lower-case character to indicate aromaticity, but also brackets to indicate non-standard number of bonds if required (pyridine derivative complexes are the most frequent case). This causes trouble with some software (see “From CIF to SMILES” section) that does not accept extra bonds for aromatic atoms, but we still think this is the most adequate representation.

**Fig. 2:**
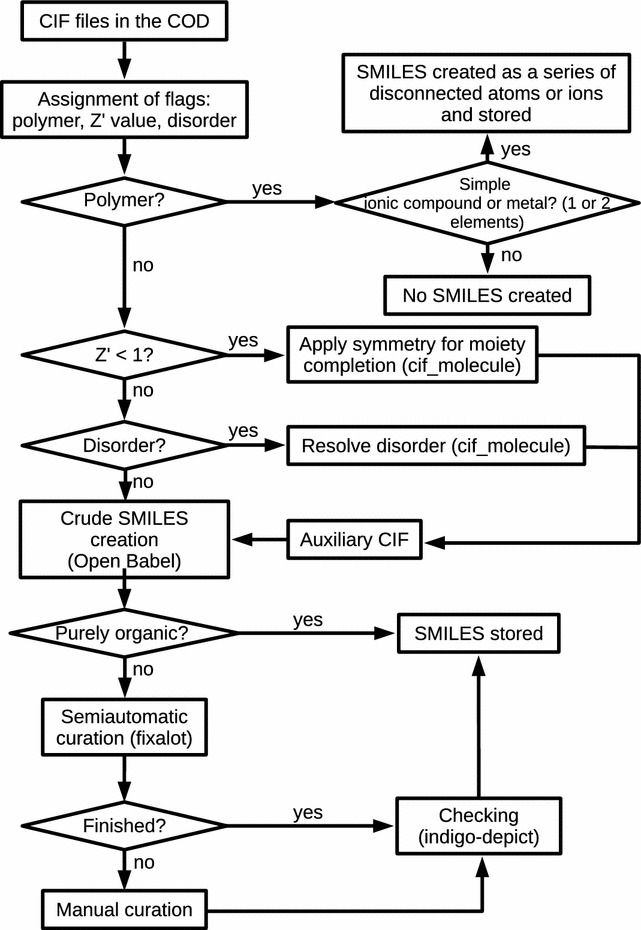
Scheme displaying the steps involved in obtaining the SMILES strings from the CIF files in the COD

A more troublesome situation is bidentate (or polidentate) ligands in which a donor atom acts in a neutral form and the other one acts in an anionic form. When this happens, we may face a delocalised bond scenario which is not covered by the SMILES specification. This happens when two resonance forms exist with the “neutral” and “anionic” atoms interchanging their roles, possibly “moving” the $\pi$-bonds between them. In these cases, we have chosen to represent only one of these resonance structures; this should not cause any trouble if both structures are equivalent (examples: chelating carboxylate or acetylacetonate), but we leave out one of the forms otherwise. Table 2 and Fig. 1b show several examples of our treatment of coordination compounds.

#### Boranes

These compounds provide an example of polycentric bonds, which cannot be properly represented in SMILES format. Our choice is to include bonds from each atom in the cage (boron or carbon or any other element that may be present) with all its neighbours at a similar distance into the SMILES representation. This implies a large number of bonds (typically six for each B or C atom), but we think this is the best approximation of the molecular geometry using the SMILES syntax. As the atoms do not have the standard valences, brackets should be always used and hydrogens (terminal or bridging) should be indicated explicitly. An example of a *closo*-dicarbadodecaborane with its corresponding SMILES is displayed in Fig. 1c.

#### Metal carbonyls

This is a family of compounds found very frequently in the COD having a bonding scheme that also falls out of the VBT. The bond order in free CO is 3, according to basic molecular orbital theory, but this bond order decreases when CO bonds to a metal atom, metal-carbon bond order is also higher than one, both actual bond orders depending on which is the metal and the presence of other ligands. According to this, the two most sensible representations for a monodentate metal carbonyl are [M]=C=O or [M]C#[O] and we have chosen the second one, since we think that, in general, it fits somewhat better with experimental values of IR frequencies or bond distances, even if it implies a non-standard valence for the oxygen atom. On the other hand, when the CO molecule bridges two metal atoms, the C-O bond order decreases further and we have chosen to regard this bond order as two, hence we consider a bridging metal carbonyl as a formal analogue of an organic ketone. Metal isocyanides are treated in a similar way to metal carbonyls.

#### Organometallics

These compounds are considered a particular case of coordination compounds so, in principle, the same rules indicated above apply here. As is the case for boranes, polycentric bonds cannot be properly represented by SMILES and bonds of the metal atom with each of the carbon atoms in a polyhapto ligand are included in our representation. For example, an alkyl derivative is formally a complex with an anionic ligand and therefore no brackets are required for the carbon atom, an $\eta ^2$ or $\eta ^4$ alkene ligand is considered neutral so that brackets and explicit hydrogens are required for all involved carbon atoms, an $\eta ^3$ ligand is considered monoanionic, so one of the terminal carbon atoms is written without brackets whereas the other two involved carbon atoms are written with brackets and forming a double bond, thus representing only one of the two possible resonance forms, all this following the same guidelines indicated above for coordination compounds.

The most frequently found type of organometallic compounds are metallocenes. These compounds are regarded by chemists as aromatic, so lower-case characters are required for the binding carbon atoms; as they do not use their standard valence, brackets and explicit hydrogens are mandatory. This representation is not honoured by some cheminformatics software (see “From CIF to SMILES” section) that does not admit the possibility of extra bonds for aromatic atoms, but we think it is the most sensible one from the human point of view. See examples of organometallic SMILES in Table 3. A compound involving carbonyl ligands and an $\eta ^6$ linked benzene ring with its SMILES is displayed in Fig. 1d.

Many other kinds of interactions appear in organometallic compounds which do not fully fit the general bonding ideas indicated in the previous paragraphs. Therefore, it is sometimes rather difficult to clearly establish which atoms of the ligands are bound to the metal and to decide which bond order scheme suits a given compound the best. In such cases an ad hoc individual choice is made, sometimes after consulting the original publication.

#### Metal–metal bonds

There is no clear criterion to distinguish when the interaction between two neighbouring metal atoms in a crystal should be considered a chemical bond. A distance criterion does not suffice, since a short distance may be imposed by other present species (for example bridging supporting ligands). A clear interaction with no supporting ligands is usually considered as bond (typical case, polynuclear metal carbonyls) and atoms at very short distances (below 2.85 Å for contacts between transition metal atoms) are also considered bonded whereas values higher than 3.0 Å with supporting ligands are disregarded. For borderline cases, we tend to accept author’s criterion, expressed as the presence of the corresponding _geom_bond_distance data item in the CIF file. No attempt is made to guess the metal–metal bond order. Consideration of the existence of metal–metal bonds usually leads to complicated SMILES in cluster compounds.

#### Formal charges

Generally speaking, we avoid the assignment of formal charges, since this implies, in many cases, assumptions about the oxidation numbers of atoms or charge distribution within chemical moieties that are not always obvious, especially if we want to make such assumptions in a more or less automated way. However, we do assign formal charges for very simple species or for moieties where the location of the charge is well established by widely accepted chemical conventions. This includes isolated charged atoms (halides, alkali cations) and simple anions, like oxoanions or haloanions: for these anions, the formal charge is assigned to the terminal atoms of the species and, in the case of oxoanions, the bond with the central atom is considered single for charged oxygens and double for non-charged ones. We must note here, however, that in some important databases like ChemSpider [16], ChEBI [25] and PubChem [24] haloanions are represented with the negative charge located in the central atom. In order to improve data cross-referencing we have also built an alternative representation of the COD entries containing simple haloanions with the negative charge assigned to the central atom instead of the halogen as explained in “Results and discussion” section.

**Table 1:** Example of aromatic or non-aromatic choices made for the representation of several compounds with arguable aromaticity

Pyrrole	c1ccc[nH]1
Thyophene	c1cccs1
Cyclopentadiene	C1=CC=CC1
Cyclopentadienyl anion	[cH-]1cccc1
Cyclopentadienone	c1(=O)cccc1
2-pyridone	c1(=O)[nH]cccc1
Uracil	C1(=O)NC(=O)NC=C1
Pyridine oxide	c1ccccn=O
Quinone	O=C1C=CC(=O)C=C1
Anthraquinone	c12ccccc1C(=O)c1ccccc1C2(=O)
9-Methylene-fluorene	c12ccccc1C(=C)c1ccccc12
Imidazolidene metal carbene	[Ni]=C1N(C)C=CN1C
$\hbox {C}_{60}$	c12c3c4c5c1c1c6c7c2c2c8c3c3c9c4c4c%10c 5c5c1c1c6c6c%11c7c2c2c7c8c3c3c8c9c4c4c 9c%10c5c5c1c1c6c6c%11c2c2c7c3c3c8c4c4c 9c5c1c1c6c2c3c41
A tetramethyl derivative of C$_{60}$	C12(C)C3C4(C)c5c1c1c6c7c2c2C8(C)C=3C3 (C)c9c4c4c%10c5c5c1c1c6c6c%11c7c2c2c7c 8c3c3c8c9c4c4c9c%10c5c5c1c1c6c6c%11c2c 2c7c3c3c8c4c4c9c5c1c1c6c2c3c41

**Table 2:** Example of the representations chosen for different kinds of coordination compounds

An ethylenediamine complex	[Ni]1[NH2]CC[NH2]1
A phosphane complex	[Au][P](c1ccccc1)(c1ccccc1)c1ccccc1
A water complex	[Zn]([OH2])([OH2])([OH2])([OH2]) ([OH2])[OH2]
A phenolate (anionic) complex	[Co]Oc1ccccc1
Dichloridebis(pyridine) copper(II)	[Cu]([n]1ccccc1)([n]1ccccc1)(Cl)Cl
An imidazole complex (neutral)	[Mn][n]1c[nH]cc1
An imidazolate complex (anionic)	[Mn]n1cncc1
Bidentate acetate moieties	[Cd]12([O]=C(O1)C)[O]=C(O2)C
Acetylacetonate complex	[Gd]1[O]=C(C)C=C(C)O1
Imino-enolate or amido-cetone complex (non-equivalent resonance forms)	[Cr]1[N](c1ccccc1)=C(C)C=C(C)O1 or [Cr]1N(c1ccccc1)C(C)=CC(C)=[O]1

**Table 3:** Example of the representations chosen for some organometallic compounds

Tetracarbonyl nickel	[Ni](C#[O])(C#[O])(C#[O])C#[O]
A compound with bridging carbonyls	[Co]1(C#[O])(C#[O])(C#[O])C(=O)[Co] (C#[O])(C#[O])(C#[O])C1=O
An alkyl derivative	[Pb](CC)(CC)(CC)CC
A $\eta ^2+\eta ^2$ ligand	[Rh]123[CH]4=[CH]1CC[CH]2=[CH]3CC4
A $\eta ^4$ ligand	[Ti]123[CH2]=[CH]1[CH]2=[CH2]3
A compound with $\eta ^3$ ligands	[Pd]1234(C[CH]1=[CH2]2)C[CH]3=[CH2]4
Ferrocene	[Fe]12345678([cH]9[cH]1[cH]2[cH]3[cH]4 9)[cH]1[cH]5[cH]6[cH]7[cH]81
A $\eta ^5$-indene ligand	[Zr]1234[cH]5[cH]1[cH]2[c]13[c]45cccc1
A $\eta ^6$-p-cymene ligand	[Ru]12345[c]6(C(C)C)[cH]1[cH]2[c]3(C) [cH]4[cH]56

Regarding organic moieties, charges are assigned to carboxylates, phenolates (if not bound to a metal) and also to ammonium/phosphonium groups. No formal charge is assigned in coordination compounds, neither to the metal atom nor to the ligand.

#### Ionic compounds

The ionic bond is intrinsically a non-directed bond and, because of it, the concept of chemical connectivity becomes rather diffuse when ionic compounds are involved. For example, in sodium chloride each ion interacts with all of its neighbours and trying to define the chemical connectivity in a NaCl crystal is beyond SMILES format capabilities, as stated above. Defining such connectivity even for a small region of the crystal would be a rather difficult task resulting in a quite complicated and almost useless SMILES string. Hence, this compound is represented by just a couple of disconnected ions: [Na+].[Cl-].

The situation is, however, not always as simple as that. We can find ions that are not monoatomic, with covalent bonds inside them and also bonds with intermediate character between covalent and ionic and we need to establish cases in which we are going to regard the ions as disconnected species and when we will try to keep the bonds, maybe defining a polymeric species.

Monoatomic anions are not usually a problem, since they are either clearly disconnected from the rest of the structure or clearly coordinated to a single (or a very limited number) of metal atoms. On the contrary, ionic interactions of monoatomic cations usually take place with a larger number of neighbours, sometimes belonging to different moieties and thus frequently generating extended ionic frameworks. We have chosen to represent such situations as disconnected ions for alkali and alkali-earth cations: for these elements connectivity is only included in the SMILES in cases when non-polymeric moieties are generated (a typical case: complexes with crown ethers), leaving these elements as monoatomic non-connected cations ([Na+]., [Ca+2].,…) otherwise. On the other hand, connectivity is retained in most cases for transition and post-transition cations, for which bonds have a more covalent character; for these elements, disconnected ionic SMILES are only used for extended structures involving only the metal cation and simple inorganic anions.

Regarding metallic crystals, no attempt has been made to get the chemical connectivity. Metals are represented as isolated atoms.

#### Stereochemistry and chirality

The OpenSMILES specification includes rules for specifying the stereochemistry around a given atom, including tetrahedral, square planar, trigonal bipyramidal and octahedral environments. Nevertheless, we are actually including only stereochemical information for the simplest and the most useful case, namely for the tetrahedral centres, using the “@” and “@@” symbols for the two possible configurations around an asymmetric tetrahedral atom.

In order to correctly mark the tetrahedral centers the space group must also be examined. If the space group is a Sohncke group (i.e. it contains only symmetry operators of the first kind, namely translations, rotations and screw rotations), all moieties in the crystal have the same configuration as in the asymmetric unit, so the stereochemical information of this asymmetric unit is representative of the whole sample and no further intervention is required. But if we have a chiral species in a space group that contains improper rotations or reflections (a non-Sohncke group), this means that the crystal is racemic and we must reflect that in the built SMILES. If the moiety contains a single chiral centre (thus, there is only a single “@” or “@@” in the SMILES string), the simple elimination of the chiral mark suffices: according to the SMILES specification, the absence of the chiral marks means “unspecified stereochemistry” which we may reinterpret as “both enantiomers present”. On the other hand, if more than one chiral mark is present in a given moiety, removing the chiral marks also eliminates the information about the relative configuration of the chiral centres, so we think that a more correct approach in these cases is to explicitly write both enantiomers, the second one created just by inverting all the “@” and “@@” present in the first, even if this means doubling the length of the SMILES string. Figure 1e shows the SMILES string for an entry corresponding to a chiral crystal containing just one pure enantiomer whereas Fig. 1f shows another entry corresponding to the related racemic crystal. A special situation are non-chiral moieties containing chiral centres (because each asymmetric tetrahedral centre has its mirror image at the other side of the moiety), the so-called meso forms in the usual language of organic chemistry: for these species, writing the moiety once is sufficient, since it is identical to its enantiomer.

### Building the molecule

A CIF file typically contains a table with the crystal coordinates of the atoms that make up the asymmetric unit. In the simplest case, this list of atoms also makes a chemically acceptable representation of the compound. From this point onward we are referring to such representation as the “molecule”, i.e. the set of atoms that a chemist usually includes when he/she writes the structural formula of a given compound and that may consist of one or more separate moieties, charged or not (at this moment, we are thinking only of finite moieties, we will consider polymers in subsequent paragraphs). The SMILES string associated with an entry in the COD should contain the involved molecule. The procedure that we have designed to obtain such strings from the original CIFs is summarised in Fig. 2 and explained in this section and the next.

In some CIFs, the asymmetric unit and the molecule are the same thing and we can build the molecule by just using the list of atoms as they appear in the CIF, but this is not always the case. It is possible that some moiety is placed on a crystallographic symmetry element and we need to apply that symmetry element to the asymmetric unit to obtain the whole molecule. We can also find that there is more than one molecule in the asymmetric unit and we will obtain a SMILES with repeated molecules if we treat the list of atom “as is”: a SMILES with repeated molecules is probably harmless for many applications (for example, for substructure searches), but is not the best representation of the compound.

A more serious problem is disorder. One kind of disorder is when a part of a molecule may be placed in two or more alternative positions, with more than one entry for a given chemically unique set of atoms; the occupancy factors of these entries should add up to one. In these cases, the easiest approach is to consider only one of the possible images of the disordered portion of the structure and ignore the other. Another type of disorder is when some moiety (typically solvent) is present only in a fraction of the equivalent positions in the crystal, the other being empty: the SMILES specification does not allow for fractional presence of moieties, hence the amount of this solvent molecules must be “rounded up” in the SMILES string. Yet another kind of disorder is when two or more different chemical species share the same region of the asymmetric unit, so the crystal is actually a mixture of more than one chemical compound. This is rather infrequent for synthetic materials, but quite common for minerals. For these cases, we usually ignore the components with very low occupancy (say below 0.1) and try to explicitly write all of the remaining species.

A further possible reason for discrepancy between the listed atoms in the asymmetric unit and the molecule is the absence of atoms not located crystallographically, but that are known to exist. We find this situation frequently for hydrogen atoms: in many cases, their presence can be deduced from the geometry of the rest of the molecule or even from simple chemical common sense, in these cases we try to add these missing hydrogens. This involves for example apparent isolated oxygen atoms that almost certainly represent water molecules. Another important case are solvent molecules that have been removed by the SQUEEZE program. As the nature of such removed moieties is usually very hard to guess using only the information in the CIF, these species are simply left out.

To address all these problems, the first step made for the CIFs contained in the COD is to perform some simple checks to establish three flags for each entry. Firstly, the value of Z’ (number of molecules in the asymmetric unit) is determined by comparison of the Z value given by the authors in the CIF with the number of symmetry equivalents of the general position in the unit cell, this Z’ value being stored in the COD MySQL database. The first flag indicates whether Z’ is lower than one, one or higher than one. A check is also made to search for disorder, if at least one of the atoms has an _atom_site_occupancy parameter lower than one, the entry is flagged as disordered using the second flag. Another check is to search for bonds between atoms of different asymmetric units, looking at the _geom_bond_site_symmetry_2 parameter for all listed bonds, if we do not find anything different than “.” or “1_555”, we infer that all bonds are within the asymmetric unit, otherwise we can either have a polymer or a moiety placed in a symmetry element, this information being stored in the third flag.

The values of these flags guide the further treatment of the file: when we find Z’ = 1 without disorder and without bonds between atoms in different asymmetric units, the entry goes directly to the SMILES generation phase (“From CIF to SMILES” section). Other entries are classified according to their assigned flags, so that the entries with similar problems are jointly processed in the same way.

Many of these problems can be solved by using the program cif_molecule, included in the *cod-tools* package [26]. The program is invoked with the options –one-datablock-output (all moieties are contained in the same output block), –geom-bond-output (_geom_bond_* data items are included in the output so they can be used afterwards) and –preserve-stoichiometry (the proportion between the present moieties reflects the stoichiometry of the compound). The features of this program and the implemented algorithms have been previously described [27]. The output of the program is a CIF file containing the list of atoms that should be included in the representation of the compound. The option –split-disorder-groups (which is on by default so it is not necessary to explicitly specify it) is useful in structures with disorder of the first kind (as described above), producing separate CIF blocks for each component of the disorder, the first of these blocks being that with the largest sum of atom occupancies. This block may be taken to generate the SMILES, just ignoring the rest. The file obtained in this way corresponds to the *P 1* representation of the crystal, therefore, containing all atoms for contiguous molecules. Such representation is required in further steps.

Polymeric species are possibly the most difficult challenge for the definition of what we consider as a “molecule”. As stated above, this kind of compounds cannot be faithfully represented using the SMILES format, we need to acquiesce to represent just a finite portion of the polymer, which should contain a number of connected monomeric units that suffices to display the most important features of the atomic connectivity.

In a chain-like (1-D) polymer, the presence of just two connected units is enough to complete the coordination sphere of at least one instance of every unique atom. If possible, each edge of the chosen set of atoms should involve just one pending chemical bond. More difficult is choosing our “molecule” for 2-D and 3-D polymers, a possibility is to include in our representation the minimum set of monomers that close a ring of the polymer, even if this may leave out important features of polymer topology, specially in the 3-D case, and not being clear if every unique atom will have a complete coordination environment. At present, we lack an automatic way of building the chosen sets of atoms, which makes polymeric compounds the category of COD entries with least available SMILES. Using cif_molecule with the “–max-polymer-span 1” option yields a portion of the polymer that is perhaps too large in most cases. The few polymeric compounds with a defined SMILES string at the moment of writing this article are 1-D polymers for which the second repeating unit has been added by applying the involved symmetry operation to the asymmetric unit once. The polymeric nature of a compound and its dimensionality are detected by cif_molecule and recorded in the output using the _cod_molecule_is_polymer and _cod_molecule_polymer_dimension data items.

The stoichiometry of the compound is retained in the procedure of building the auxiliary CIF file that includes all atoms of our “molecule” (the –preserve-stoichiometry option of cif_molecule), so that this auxiliary CIF will contain a chemically faithful representation of the whole compound. Nevertheless, there are some cases, mostly related to fractional (solvent):(main compound) ratio, in which the stoichiometry is not fully preserved in the final SMILES, as indicated in the next section.

### From CIF to SMILES

Once we have a CIF file containing the atoms we want to include in the representation of our compound, the next step is to get the SMILES string corresponding to that set of atoms. To perform this task, we are using *Open Babel* [28], a package of free software for interchange between chemical formats. In our case the input is CIF and the output is SMILES. *Open Babel* has been chosen because it is open source, it is continuously and actively updated by its development team, has a large user community, it can be invoked from the command line and it delivers satisfactory primary results even if these need to be inspected and curated as indicated below.

At the moment of writing this paper *Open Babel* version 2.4.1 is the latest one available on the official website [29], however, we have been consistently using the 2.2.3 legacy version since the start of our endeavour and there are several reasons that justify this choice. Firstly, it ensures the uniform representation of the entries—a quality that could be compromised while switching from one *Open Babel* version to another. An even more important reason, however, is a set of changes dealing with the perception of aromaticity and the addition of missing hydrogen atoms that have been introduced in versions later than 2.2.3. The changes in question probably improved the treatment of organic compounds, but seriously broke the handling of the inorganic ones. A very common kind of compound that displays this problem is the pyridine-like metal complexes which are correctly described with the [Cu][n]1ccccc1 SMILES when using version 2.2.3, but are grossly misrepresented as the [Cu]N1CCCCC1 SMILES (full hydrogenation of pyridine, transforming it into piperidine!) when using later versions. Finally, the more recent versions of *Open Babel* do not seem to honour the -aB options (that consider bonds listed in the _geom_bond_* CIF data items, see below) thus rendering the software unsuitable for our needs. The source code has been downloaded from the *Open Babel* website and has been compiled and installed in computers running Ubuntu versions ranging 10–16 on both 32 and 64 bit architectures.

*Open Babel* takes the atoms as they appear in the input file, without generating extra atoms by symmetry or discriminating disordered components, so the input CIF is not always the original file present in the COD, but, in many cases, it is the result of applying cif_molecule to that file as described in “Building the molecule” section. In principle, just performing “babel –title xxxxxxx xxxxxxx.cif -osmi> xxxxxxx.smi” will yield a file containing a single line, with the output SMILES string and the COD ID of the entry (xxxxxxx in the previous command) separated by a tab character.

*Open Babel* ignores atomic occupancy factors, so partial solvent occupancy due to disorder is “rounded-up” as indicated in the previous section. Likewise, solvent content is also rounded-up when solvent moieties are placed on symmetry elements, resulting in a fractional (solvent):(main compound) ratio, (typical case: hemi- or sesquihydrates), avoiding the duplication of the main species. Such “rounding-up” procedure is applied only to simple moieties of compounds usually used as solvents; in general, nothing more complex than a toluene moiety is ever considered as “solvent”. Obviously, for these cases, the stoichiometry is not strictly preserved in the final SMILES.

For performance reasons, the number of bonds formed by a given atom is limited in the internal *Open Babel* representation, the limit being hard-coded into the *Open Babel* source code and because of this, some atoms linked to many neighbours may appear with lacking bonds in the output. Examples of this are boranes and carboranes (boron and carbon typically forming six bonds), many kind of organometallic compounds as metallocenes (both metal and carbon atoms frequently form a number of bonds above *Open Babel* limit), f-block element complexes (high coordination numbers), etc. Because of this, we usually get better results if we include the “-aB” option in babel command line, this meaning adding the bonds included in the CIF to those detected by *Open Babel*. Nevertheless, there are exceptions to this since the authors sometimes describe distances in the CIF file that are interesting to them (metal–metal weak interactions, hydrogen bonds listed in the wrong place, etc.), but that should not be considered bonds and hence should not be taken into account when building the SMILES. Because of it, we compute the SMILES from the CIF both with and without the -aB option; if both results are identical, any of them is used to create the SMILES string, if they are not, both results are presented on the screen so that the choice is made by an human operator (the best option is quite obvious in most cases and the choice is made in a couple of seconds). The result is what we call “crude SMILES” hereafter. This process of crude SMILES creation is facilitated by the helper Bash (terminal interpreter language supported by most Linux and BSD operating systems, version 4.3.11 has been used through this work) script smi_create from the *smiles-scripts* package (version 0.1.0, released under the GPL2 free software license, available at the COD Web site [30] and including also other scripts mentioned in this section).

These crude SMILES are usually a good image of the chemical species inside the crystal, but they frequently contain small imperfections or do not completely conform to the conventions described in “Format and conventions” section. Because of it, the crude SMILES need to pass through a curation process before they are included into the COD public repository. The SMILES creation and curation process is performed on subsets of around 1000 entries, each subset being made up of entries in a given range of COD IDs and with the same flags (previously established as indicated in “Building the molecule” section). The corresponding set of CIFs, either the original ones or those generated by the cif_molecule (depending on the flag values) are used as input for *Open Babel* to get the crude SMILES. The first curation step of this set is to separate the files that do not contain square brackets, this meaning that the corresponding compounds contain only elements of the organic subset acting with their standard valence (mostly purely organic compounds); these SMILES receive just a quick look and, if nothing wrong or unusual is detected (which happens in most cases), are directly accepted as final SMILES.

After this, there are families of compounds for which *Open Babel* does not produce an output conforming to our conventions, but for which a simple find/replace routine may produce a more acceptable result. For example, the crude SMILES for a monodentate metal carbonyl is systematically given as [M]([C][O]) whereas we want it to be [M](C#[O]), such a change being easily automatically made by a script. Nevertheless, the result must be inspected by a human expert since, for example, a methanol molecule with missing hydrogen atoms could be mistaken for a carbon monoxide moiety. The script fixalot from *smiles-scripts*, written in Bash, performs a great number of such automatic changes, involving species frequently found in crystal structures such as perchlorate, nitrate, coordinated azide, metal carbonyls, metal isocyanides, haloanions, some common coordinated solvents (MeCN, DMF, DMSO, etc.), imines with a wrong single C–N bond, coordinated phosphane ligands, hydrogen-less water molecules, etc. This script is continuously updated by adding chemical species once they start to appear frequently in the database, as long as the curation can be made with just a find/replace routine. The Additional file 1 includes a table with a list of the changes performed by the script at the moment of writing this article. Every time the script performs a change, the original string and the modified one are displayed on the screen and a human editor quickly decides if the change is correct or not. The final curated SMILES is also displayed to decide if it already qualifies as a “final result” to be stored or, otherwise, passed to the manual editing phase.

Reaching this point, the entries that have not yet been cleared out enter the manual editing phase. Bash scripts have been designed to extract groups of entries likely to present the same kind of problem because it is easier and faster for the human editor to modify SMILES strings of similar nature in the same way. One of the most outstanding examples of this are metallocenes, that are readily identified and separated performing an *Open Babel* search, but that cannot be easily fixed in an automated way to conform the conventions indicated in “Format and conventions” section. Something very similar can be said about carboranes and boron compounds in general. Other categories of compounds that are identified and separated for manual inspection and possible fixing are those containing C, N, O or S atoms inside square brackets, possibly with attached explicit H, but without chiral marks, indicating that these atoms act with apparent non-standard valences, which may be due to an error in the SMILES that should be fixed, coordination to a metal atom (and hence being correct) or missing or spurious H atoms among other possible reasons.

There are many kinds of manual fixes that need to be applied to the crude SMILES in order for them to become suitable for the inclusion in the COD repository. Frequent cases are removal of spurious bonds, like metal–metal weak interactions or the bond of a metal to the opposite atom in a four member chelate ring (most frequent case, the carbon atom of a bidentate carboxylate group), wrong bond orders frequently coupled with spurious H atoms (typical examples include coordinated imine and carbonyl groups), resonance forms that are clearly not the most adequate ones, etc.

An important case are chiral moieties, easily identified by the presence of the “@” character. The space group for the corresponding entries is checked to flag the crystal as chiral or not-chiral and to proceed afterwards accordingly to the guidelines outlined in “Format and conventions” section. Detection of the “@” character, checking of space group, elimination of chiral marks and enantiomer generation are automatically performed by Bash scripts when applicable. Non-chiral moieties with chiral atoms, when detected, cause the enantiomer generation step to be skipped.

In the process of manual editing, it is necessary to use visualisation programs (such as winortep [31] or the jav viewer included in the *SIR2014* package [32]) for a large number of entries, to be sure of the nature of the involved chemical entities, in a few cases (fortunately, very few) even the original publication has to be checked. Whenever any manual editing is performed, the resulting SMILES is represented using the indigo-depict program [33], thus checking for syntax errors and many semantic mistakes, before being considered acceptable. Just as a matter of curiosity, the longest SMILES string created so far is that for the COD entry 1,513,721, a complex yet discrete cluster with 52 metallic atoms [34] and a SMILES string of 2778 characters.

As previously indicated, the concept of “chemical connectivity” is rather fuzzy for purely ionic and metallic crystals and our choice for representing them is just to write a list of disconnected atoms or ions, useful for identification purposes. Most of these entries are flagged as 3-D polymers and therefore they have not entered the above described procedure for molecule building and *Open Babel* SMILES creation. For the most simple cases (mostly pure elements or binary compounds which are easy to identify in the database), SMILES have been created by just writing the corresponding list of disconnected atoms trying to keep the stoichiometry. The procedure needs to be extended to other frequent compounds such as mixed oxides, metallic salts of simple anions, etc.

## Results and discussion

After curated SMILES have been generated for a given subset of entries, the resulting single-line files are stored and made publicly available at the COD Web site  [35]. Apart from this collection of small files, all SMILES are also stored in a large single file (one entry per line) named allcod.smi, also available in the repository. This file is regenerated each time new SMILES are added and a fingerprints file (allcod.fs) is created from allcod.smi, available to be used for fast searches. The process of creating allcod.fs from allcod.smi is also useful as yet another check to detect possible syntax errors, since the presence of a syntactically wrong SMILES in allcod.smi interrupts the creation of allcod.fs and the moment in which the process is interrupted directly points to the position of the offending entry.

At present (March 2018), allcod.smi contains 160 697 curated SMILES that represent 41.0% of the 392 315 COD entries. The files allcod.smi and allcod.fs are available to perform substructure searches in them or, in other words, in the subset of the COD for which SMILES have been defined. Searches can be done using *Open Babel* with any of these two files: if we use allcod.smi, a typical search will take several minutes (on a PC with a 1.6 GHz 4-core processor) and so, we call it the “slow search” and if we use allcod.fs, the search will take only a few seconds, therefore we call this the “fast search”. The query for a fast search must be a valid SMILES string whereas the query may be written in the much more versatile SMARTS (SMILES Arbitrary Target Specification) language [36] for a slow search, this versatility compensating in many cases the long time required for this kind of search. The command for performing the slow search is:

babel allcod.smi -osmi -s "$SMARTS" | cut -f2

where $SMARTS is the SMARTS pattern to be searched for, the output being a list of matching COD IDs. For a fast search just replace allcod.smi by allcod.fs using a valid SMILES as a query instead of a SMARTS pattern.

An alternative version of allcod.smi is also being built moving the formal charge of simple haloanions from the halogen atoms to the central atom, since this representation is preferred in other data sources like PubChem. To build this alternative representation, we first select those entries from the allcod.smi file that have a B,P,S,As,Al,Bi,Sn,Si or Sb “central” atom with at least three halogen atoms attached to it, and place it into the allcod-hal.smi file using obgrep governed by a GNU Make Makefile script. Afterwards, the allcod-hal.smi file is filtered through the cdkrecharge program to produce the allcod-alt.smi file. The cdkrecharge program uses the CDK toolkit [37] to parse SMILES, handle charges and write out the resulting new SMILES. The cdkrecharge algorithm, implemented in Java, picks distinct moieties from the parsed SMILES structure that have only 2 distinct atom types. One atom type must be the above mentioned “central” atom and another type must be a halogen, and the total charge of the moiety must be negative. For such moieties (haloanions), the cdkrecharge marks all halogens as neutral, puts all negative charge on a single “central” atom, and, if the total charge of the processed moiety did not change, replaces its representation by a new SMILES sub-string generated from a moiety with redistributed charges; if any of the mentioned checks fails, the moiety is left unaltered. This procedure transforms, for example, a [P](F)(F)(F)(F)(F)[F-] SMILES component into a [P-](F)(F)(F)(F)(F)F representation. The Makefile used for this conversion is available in the COD repository smi subdirectory, and the cdkrecharge program is included in the *smiles-scripts* package. When searching for haloanions using SMILES from external sources (such as PubChem), it is recommended to either use uncharged forms or to search both allcod.smi or allcod.fs and allcod-alt.smi files, to make sure that the molecule of interest is found regardless of charge representation used (the three files are provided in the COD repository).

At present, only the fast search is implemented in the COD website and its mirrors, since the long time span taken by the slow search poses problems to Web performance. The query may be directly introduced by the user as a SMILES string or built using a graphical user interface based on JSME [38]. Alternatively, the user may download allcod.smi to his/her own computer and install *Open Babel* (version 2.2.3 is recommended as previously stated, more recent versions being more prone to miss hits, specially when using inorganic queries) to perform slow searches or even to use other search engines instead of *Open Babel*. And, of course, this SMILES collection may be used for any other purpose different from substructure search. The SMILES strings are also included in the web information card for the corresponding COD entries (directly accesible at http://www.crystallography.net/xxxxxxx.html, xxxxxxx being the COD ID).

When using *Open Babel* for the search purposes (either the fast or the slow mode) it must be taken into account that for some compounds the internal software’s representations of the moieties may be different from the strings stored in allcod.smi (hence, not conforming to the conventions indicated in “Format and conventions” section). Probably, one of the most outstanding families of compounds for which this happens is metallocenes that appear as non-aromatic in the internal representation, so we need to write the query using “C” for the atoms linked to the metal instead of “c”. In compounds with arguable aromaticity and in those with two or more reasonable non-equivalent resonance forms, it is advisable to perform searches considering all possibilities to avoid missing results. Formal charges are better omitted, since a non-charged query may hit charged species, but not the other way round.

A test of the validity of the obtained SMILES has been performed by generating SMILES strings by applying the OPSIN v2.3.0 program [39] to derive chemical connectivity from systematic chemical names of the COD entries. On 2017-07-14 COD contained 122,513 entries with known systematic chemical names (defined by CIF data item _chemical_name_systematic). Most of these entries originate from the supplements of *Acta Crystallographica* Sections C and E, *Organic Letters*, *Cryst. Eng. Comm.*, *Inorganic Chemistry* and *Dalton Transactions*. We have already generated curated SMILES for 30,109 of these entries, therefore this set of entries was selected for the validity test. CIF markup for strings in italics (<i>...</i>) was stripped from chemical names before processing them with OPSIN. Both curated and OPSIN-derived SMILES were then canonicalised using *Open Babel* 2.2.3. For 19,475 entries (65%) in the set, corresponding canonicalised SMILES were found to be identical, other entries have been automatically analysed to establish the reasons of the discrepancy. The results of such analysis are displayed in Table 4, table rows being ordered in ascending importance of the corresponding discrepancy type. Entries associated with several discrepancy types are treated as representing only the most important discrepancy type (thus only appearing in the table row closer to the bottom).

A share of 35% of the discrepancies may appear as too large at first sight, but most of these apparent differences are not attributable to misrepresentations of the involved compounds. We can see a large number of entries without information about double bond or chiral centre configuration in OPSIN output because such information was not included by the authors in the compound name in the CIF. In a small number of cases, the chirality information is missing in the SMILES of the current study, this happening mostly for racemic entries for which the compound name in the CIF specifies a single enantiomer and also for compounds containing sulfoxide groups, since OPSIN sometimes generates chirality information for them, this not complying with the SMILES specification. A numerous group is quoted in the “different representation of racemates” row, these are racemic crystals with more than one chiral centre in each molecule that we represent by explicitly writing both enantiomers (see “Format and conventions” section) whereas OPSIN output contains just one molecule, either without chirality information or describing just one of the enantiomers.

The small group “different numbers of explicit H” includes entries with different tautomeric choices for H atoms riding on N atoms in heterocycles (imidazole-like compounds). The largest set in the table is that involving different representation of nitro groups (including nitrate anions), which are depicted as [O-][N+](=O) (or [N+](=O)[O-]) by OPSIN, whereas our method uses O=N(=O) or N(=O)=O. Other groups like diazo (including azides) and N-oxides display a similar behaviour. The row “different charge settings” lists entries with different distribution of formal charges, but that essentially are alternative representations of the same thing. Therefore, we consider that the discrepancies included in this row and all rows above it are not significant, the results from OPSIN and from our procedure being essentially identical. Considering this, the share of identical entries increases to 86.67%.

The number of remaining entries is still high enough to deserve a deeper investigation. As checking them one-by-one would be far too cumbersome, we have performed a sampling procedure. We have taken a number of entries from each row (typically 25, but taking all of them for rows with a small number of entries and 50 for the more populated last one) and compared both canonicalised SMILES with a representation of the original CIF. 481 entries have been analysed in this way and we have found that, for a vast majority of them, either both canonicalised SMILES are essentially identical or our representation fits better with the real structure than that yielded by OPSIN. The most frequent situations we have found are:

- Different resonance forms in both representations defining the same chemical entity, even if the location of double bonds is not the same.
- Different formal charge assignments. Usually OPSIN is more prone to output zwitterionic structures with separated charges and our procedure is more likely to yield neutral moieties or just to ignore the location of formal charges.
- A tautomeric form deduced from the compound name in the CIF which is different from that revealed by the X-ray results, this producing a difference in the position of one or two H-atoms. The tautomer present in allcod.smi is that indicated by the crystallographic analysis so we must infer that is the correct one.
- Metal-ligand bindings missing in OPSIN representation of coordination and organometallic compounds (for example, metallocenes), probably due to missing information of binding sites in the compound name of the CIF.
- Compounds that do not form discrete molecules and that are regarded as ionic by our procedure and as molecular by OPSIN (for example, SnO$_{2}$ as [Sn](=O)=O). These compounds should be represented as disconnected ions according to the conventions stated above.
- Disagreement in solvent content: most frequent case is solvent absent from the compound name in the CIF (and hence, in OPSIN representation), in a few cases we find also the opposite (solvent not located by crystallographic analysis but included in compound name). There are also compounds with fractional (solvent):(main compound) ratios that have been rounded up by our procedure (see above) and that are stoichiometrically adjusted by OPSIN by duplicating the main species.
- Equivalent SMILES in both procedures that do not yield identical strings after canonicalisation.
- Functional groups placed in wrong places by OPSIN because of incorrect numbering scheme of organic compounds in the CIF.
- Completely wrong compound names (even absurd in some cases) in the CIF resulting in erroneous OPSIN output.

We must emphasise, nevertheless, that most inaccuracies in OPSIN output are due to wrong compound nomenclature in the CIFs and not to OPSIN malfunction. To illustrate this problem, we have listed in the Additional file 1 a small sample of entries with wrong compound names in the CIF, indicating also, for each of them, the correct name and both SMILES strings.

From 481 analysed entries we have found only 23 for which the SMILES included in allcod.smi were incorrect, this number may be extrapolated to $\sim \,160$ if the proportion of unacceptable entries is kept in each of the rows in Table 4, this meaning only 0.52% of the total of entries with systematic name information in the CIF and built curated SMILES. This is a very rough estimation, but may suffice to be confident that the real number of wrong entries in this set is clearly below 1%.

**Table 4:** Comparison of curated and OPSIN-derived SMILES

Count	%	Type
19,475	64.68	Identical
1648	5.47	Missing description of configuration around double bonds in OPSIN
17	0.06	Different number of explicit H
602	2.00	Missing chirality information in OPSIN
49	0.16	Missing chirality information in this work
1130	3.75	Different representation of racemates
2474	8.22	Different representation of nitro groups
33	0.11	Different representation of other groups
667	2.22	Different charge settings
18	0.06	Different aromaticity settings
302	1.00	Different bond orders
66	0.22	Different representation of ionic compounds
25	0.08	Missing O moieties in OPSIN
94	0.31	Different connectivity
74	0.25	Different number of rings
954	3.17	Different number of moieties
229	0.76	Different configuration around double bonds
233	0.77	Different configurations at chiral centres
50	0.17	Missing moieties in OPSIN
17	0.06	Missing moieties in this work
166	0.55	Missing C atoms in OPSIN
87	0.29	Missing C atoms in this work
190	0.63	Different stoichiometry
342	1.14	Different chemical composition
1167	3.88	Reason different from those listed above
30,109	100.00	Total

The discrepancies are listed in the table in increasing order of severity. Any entry displaying more than one discrepancy reason is included only in the category corresponding to the most serious discrepancy reason found (i.e., that closer to the bottom of the table)

## Conclusions

Work has been started to extract the chemical connectivity from entries contained in the Crystallography Open Database. SMILES format has been chosen for the representation of this connectivity and the SMILES strings are made open and freely available from the COD servers as soon as they are built. The SMILES framework was found adequate to describe a large subset of chemical moieties present in COD structures. These SMILES strings may be used to perform substructure search or for identification purposes.

The SMILES format is designed under assumption of the valence bond theory framework so the representation of compounds well explained by this theory (typically organic compounds) is usually well defined, but some conventions have been put forward to establish the way in which many inorganic and metal–organic species are going to be represented, namely fullerenes, metal carbonyls, metallocenes and other organometallics, miscellaneous coordination compounds, carboranes and other boron compounds, compounds with metal–metal interactions, etc. Ionic compounds and metals with non-directed bonds can be reasonably represented as lists of disconnected moieties.

The general procedure used for extracting the chemical connectivity from a CIF file starts with a check of the file to identify problems such as disorder, the presence of symmetry elements in the species of interest or the formation of polymeric species. Some of these problems are solved by the use of the previously published cif_molecule program. SMILES are then created by means of the *Open Babel* program package and the results are examined/curated by a combination of human editing and the use of helper scripts.

Several tests performed on the COD CIFs and on the final SMILES strings show that our procedure produces a faithful description of chemical moieties from CIFs provided by the COD. The described procedure combines both automated and manual tasks, allowing to have all SMILES strings curated by human experts, but limiting the need of human intervention only to those cases that cannot be satisfactorily resolved in an automated way. The final SMILES, even after manual editing, adhere to the OpenSMILES specification and are mostly accepted and correctly interpreted by different cheminformatics software such as indigo-depict, *Open Babel* or CDK. The generated SMILES are integrated into the COD Web site using the version control system, which ensures both data provenance and the record of maintenance history.

More than 160,000 SMILES strings have been created and made openly available in this way. We think that this result must be communicated to the scientific community even if the procedure still takes a considerable amount of human work and certainly needs to be improved and made more automatic.

## Additional file

**Additional file 1.** List of automatic changes performed by the fixalot script and examples of COD entries with wrong compound names in the CIF.
