# Supplementary material for: Using SMILES strings for the description of chemical connectivity in the Crystallography Open Database
Source: J Cheminform. 2018 May 18;10:23. doi: 10.1186/s13321-018-0279-6 (PMC5959826; doi:10.1186/s13321-018-0279-6)
Supplement: Supplementary file 1 — Additional file 1. List of automatic changes performed by the fixalot script and examples of COD entries with wrong compound names in the CIF. [file 13321_2018_279_MOESM1_ESM.pdf]

Using SMILES strings for the description of chemical connectivity in the Crystallography Open Database

Miguel Quirós, Saulius Gražulis, Saulė Girdzijauskaitė, Andrius Merkys and Antanas Vaitkus.

SUPPLEMENTARY MATERIAL

Table S-1: Automatic changes performed by the *fixalot* script (as in revision 164).

**Functional group**	**Crude SMILES**	**Fixed SMILES**
Perchlorate	[Cl]([O])([O])([O])[O]<br>[O][Cl]([O])([O])[O]	Cl(=O)(=O)(=O)[O-]<br>[O-]Cl(=O)(=O)=O
Tetrafluoruroborate	FB(F)(F)F<br>B(F)(F)(F)F	[B](F)(F)(F)[F-]<br>[B](F)(F)(F)[F-]
Nitrate	N(=O)(=O)[O]<br>O=N(=O)[O]	N(=O)(=O)[O-]<br>O=N(=O)[O-]
Hexafluorurophosphate	FP(F)(F)(F)(F)F<br>P(F)(F)(F)(F)(F)F	[P](F)(F)(F)(F)(F)[F-]<br>[P](F)(F)(F)(F)(F)[F-]
Tetraphenylborate	B bonded to 4 aromatic c	[B-]
Imidazolylborate	B bonded to 3 nn or NN groups	[BH]
Coordinated acetonitrile	[N][C]C	[N]#CC
Azide (organic o monodentate)	[N]=[N+]=N<br>N=[N+]=[N]	N#N=N<br>N=N#N
Coordinated chloride<br>(idem bromide, iodide)	[Cl-] (non isolated)	Cl
Tetrachlorometalates<br>(oxidation state +3)	[Fe](Cl)(Cl)(Cl)Cl<br>(also Al, Au)	[Fe](Cl)(Cl)(Cl)[Cl-]
Tetrachlorometalates<br>(oxidation state +2)	[Co](Cl)(Cl)(Cl)Cl<br>(also Zn, Cd, Pd, Pt, Cu)	[Co](Cl)(Cl)([Cl-])[Cl-]
Metal carbonyl	[C][O]	C#[O]
Metal isocyanide	[C][N]	C#[N]
Borane	At least one B bonded to 3+ B	[BH]
Diazo compound	[N][N]	/N=N/
Nitroso group	[N][O]	N=O
Coordinated DMF	O[CH]N(C)C	[O]=CN(C)C
DMSO	O[S](C)C<br>[O][S](C)C<br>[S]([O])(C)C<br>S([O])(C)C	[O]=S(C)C<br>[O]=S(C)C<br>S(=O)(C)C<br>[S](=O)(C)C
Organic imine	[CH][N]<br>[N][CH]	/C=N/<br>/N=C/
Coordinated imine	[CH]Nx x=(1..9)<br>[CH]N%<br>Nx[CH] x=(1..9)<br>N([CH]	C=[N]x<br>C=[N]%<br>[N]x=C<br>[N](=C
Phosphane complexes	P bonded to metal	[P]
Sulfonate	S(=O)(=O)[O]<br>S(=O)(=O)([O])	S(=O)(=O)[O-]<br>S(=O)(=O)([O-])
Interstitial water	Isolated [O]	O
Oxo groups	[O] bonded to V, Cr, Mo, W, Tc,<br>Re, U, Np, Pu	=O
Ether complexes<br>(idem thioether)	[O@H]<br>[O@@H]	[O]<br>[O]

Examples of COD entries with wrong compound names in the CIF

20 entries are listed, showing the name indicated in the CIF, the SMILES that reflects that name (obtained by OPSIN), the correct nomenclature and the SMILES deduced from atom coordinates (this work).
This is only a small sample set: many entries included in the last four rows in Table 4 display discrepancies between OPSIN output and the results of this work because of wrong compound names in the CIF. There are for sure several hundreds like these in the studied subset.

1008034
Name in CIF: Tungsten fluoride
SMILES obtained by OPSIN: F[W](F)(F)F
Correct name: Tungsten hexafluoride
SMILES in allcod.smi: [W](F)(F)(F)(F)(F)F

1505352
Name in CIF: spiro[1,3-benzodioxine-2,1'-cyclohexane]
SMILES obtained by OPSIN: C1CCC2(CC1)OCc1c(O2)cccc1
Correct name: spiro[4,4-diphenyl-1,3-benzodioxine-2,1'-(4'-phenyl)-cyclohexane]
SMILES in allcod.smi: C12(CCC(CC1)c1ccccc1)Oc1ccc(cc1C(c1ccccc1)(c1ccccc1)O2)OC

1505894
Name in CIF: vinylcyclopentan
SMILES obtained by OPSIN: C=CC1CCCC1
Correct name: 1R,2S,3R,4S-1-hidroxyethyl-1-terbutyldimethylsiloxy-3-hydroxy-4-methyl-2-vinyl-cyclopentane.
SMILES in allcod.smi: O[C@@H]1[C@H](C[C@@](O[Si](C(C)(C)C)(C)C[C@H]1C=C)CO)C

2011584
Name in CIF: 1-[4,6-dimethylthio-2H-pyrazolo[3,4-d]pyrimidin-2-yl]-4,6-dimethylthio-1H-pyrazolo[3,4-d]pyrimidine
SMILES obtained by OPSIN: CSc1nc(SC)c2c(n1)nn(c2)n1ncc2c1nc(SC)nc2SC
Correct name: 1-(4,6-dimethylthio-2H-pyrazolo[3,4-d]pyrimidin-1-yl)-3-(4,6-dimethylthio-2H-pyrazolo[3,4-d]pyrimidin-2-yl)-propane
SMILES in allcod.smi: n1n(cc2c(SC)nc(SC)nc12)CCCn1ncc2c(SC)nc(SC)nc12

2016225
Name in CIF: 3-aminopyrimidinium 3-carboxy-4-hydroxybenzenesulfonate
SMILES obtained by OPSIN: OC(=O)c1cc(ccc1O)S(=O)(=O)[O-].NN1C[NH+]=CC=C1
Correct name: 3-aminopyridinium 3-carboxy-4-hydroxybenzenesulfonate
SMILES in allcod.smi: c1(c(ccc(c1)S(=O)(=O)[O-])O)C(=O)O.c1c(ccc[nH+]1)N

2102504
Name in CIF: 1-phenyl-4-nitro-5-bromo-imidazole
SMILES obtained by OPSIN: [O-][N+](=O)c1ncn(c1Br)c1ccccc1
Correct name: 1-phenyl-2-methyl-4-nitro-5-bromo-imidazole
SMILES in allcod.smi: n1(c2ccccc2)c(C)nc(N(=O)=O)c1Br

2201456
Name in CIF: 3,5-dimethyl-2H-pyrazol-1-ium phosphonic acid diphenyl ester
SMILES obtained by OPSIN: O=P(Oc1ccccc1)Oc1ccccc1.Cc1[nH+][nH]c(c1)C
Correct name: 3,5-dimethyl-2H-pyrazol-1-ium diphenyl phosphate
SMILES in allcod.smi: P(=O)([O-])(Oc1ccccc1)Oc1ccccc1.[nH+]1[nH]c(C)cc1C

2206336
Name in CIF: Bis[2-(1-phenyl-1H-tetrazol-5-ylsulfanyl)ethoxy] ether
SMILES obtained by OPSIN: O(OCCSc1nnnn1c1ccccc1)OCCSc1nnnn1c1ccccc1
Correct name: 1,2-Bis[2-(1-phenyl-1H-tetrazol-5-ylsulfanyl)ethoxy]-ethane
SMILES in allcod.smi: C(OCCSc1nnnn1c1ccccc1)COCCSc1nnnn1c1ccccc1

2203500
Name in CIF: 2-benzyl-6-diethylamino-4-phenyl-2H-1,3,5-thiadiazine-2-carboxylate
SMILES obtained by OPSIN: CCN(C1=NC(=NC(S1)(Cc1ccccc1)C(=O)[O-])c1ccccc1)CC
Correct name: Methyl 2-benzyl-6-diethylamino-4-phenyl-2H-1,3,5-thiadiazine-2-carboxylate
SMILES in allcod.smi: S1C(=NC(=NC1(C(=O)OC)Cc1ccccc1)c1ccccc1)N(CC)CC

2209648
Name in CIF: Bis[2-(2-isopropylphenylimino)phenyl]mercury(II)
SMILES obtained by OPSIN:
CC(c1ccccc1N=C1C=CC=CC1[Hg-]C1C=CC=CC1=Nc1ccccc1C(C)C)C
Correct name: Bis[2-(2-isopropylphenyliminomethyl)phenyl]mercury(II)
SMILES in allcod.smi: [Hg](c1ccccc1/C=N/c1ccccc1C(C)C)c1ccccc1/C=N/c1ccccc1C(C)C

2212657
Name in CIF: 2-[4-(Diethylamino)phenyl]-1-ethylimidazo[4,5-f][1,10]phenanthroline
SMILES obtained by OPSIN: CCN(c1ccc(cc1)c1nc2c(n1CC)c1cccnc1c1c2cccn1)CC
Correct name: 2-[4-(Diphenylamino)phenyl]-1-ethylimidazo[4,5-f][1,10]phenanthroline
SMILES in allcod.smi:
n1cccc2c3n(CC)c(nc3c3cccnc3c12)c1ccc(N(c2ccccc2)c2ccccc2)cc1

2233448
Name in CIF: Benzoic acid--3,4-bis[(pyridin-3-ylmethyl)amino]cyclobut-3-ene-1,2-dione (1/2)
SMILES obtained by OPSIN:
O=C1C(=O)C(=C1NCc1cccnc1)NCc1cccnc1.O=C1C(=O)C(=C1NCc1cccnc1)NCc1cccnc1
.OC(=O)c1ccccc1
Correct name: Benzoic acid--3,4-bis[(pyridin-3-ylmethyl)amino]cyclobut-3-ene-1,2-dione
(2/1)
SMILES in allcod.smi:
O=C1C(=O)C(=C1NCc1cccnc1)NCc1cccnc1.OC(=O)c1ccccc1.OC(=O)c1ccccc1

2239483
Name in CIF: 5-Bromo-3-cyclohexylsulfinyl-2,4,6-trimethyl-1-benzofuran
SMILES obtained by OPSIN: Cc1oc2c(c1S(=O)C1CCCCC1)c(C)c(c(c2)C)Br
Correct name: 5-Bromo-3-cyclohexylsulfonyl-2,4,6-trimethyl-1-benzofuran
SMILES in allcod.smi: Cc1oc2c(c1S(=O)(=O)C1CCCCC1)c(C)c(c(c2)C)Br

2240483
Name in CIF: 1-Benzylsulfonyl-1,2,3,4-tetrahydroquinoline
SMILES obtained by OPSIN: O=S(=O)(N1CCCc2c1cccc2)Cc1ccccc1
Correct name: N'-(3-tolyl)-dithio-benzyl-hydrazide
SMILES in allcod.smi: S(C(=S)NNc1cc(ccc1)C)Cc1ccccc1

4331494
Name in CIF: 1,5-Diamino-1H-tetrazolium iodide
SMILES obtained by OPSIN: N[NH+]1N=NN=C1N.[I-]
Correct name: 1,5-Diamino-4-methyl-1H-tetrazolium iodide
SMILES in allcod.smi: [I-].n1(nn[n+](c1N)C)N

7008105
Name in CIF: 1-dimethylphosphino-2,2-dimethylhydrazine
SMILES obtained by OPSIN: CN(NP(C)C)C
Correct name: 1-diphenylphosphino-2,2-dimethylhydrazine
SMILES in allcod.smi: P(NN(C)C)(c1ccccc1)c1ccccc1

7013905
Name in CIF: Manganese(II)-di-chloro-2-((bis(pyridin-2-ylmethyl)amino)methyl)-4-nitrophenol
SMILES obtained by OPSIN: Oc1cc(Cl)c(c(c1CN(Cc1ccccn1)Cc1ccccn1)Cl)[N+](=O)[O-].[Mn+2]
Correct name: (2-((bis(pyridin-2-ylmethyl)amino)methyl)-4-nitrophenol)-di-chloro-manganese(II) monomethanol solvate
SMILES in allcod.smi: [Mn]12(Cl)(Cl)[N](Cc3[n]1cccc3)(Cc1[n]2cccc1)Cc1c(O)ccc(N(=O)=O)c1.OC

7106864
Name in CIF: ((1S,3S)-6,7-dimethoxy-1-phenyl-1,2,3,4-tetrahydroisoquinolin-3-yl)methanol
SMILES obtained by OPSIN: OC[C@@H]1Cc2cc(OC)c(cc2[C@@H](N1)c1ccccc1)OC
Correct name: (S-1,2,3,4-tetrahydroisoquinolin-3-yl)methanol
SMILES in allcod.smi: C1c2ccccc2C[C@@H](CO)N1

7153382
Name in CIF: rhenium cyclohexylnicotinamide
SMILES obtained by OPSIN: NC(=O)c1cccnc1C1CCCCC1.[Re]
Correct name: tri-carbonyl-(bipyridine)-(N-cyclohexylnicotinamide)-rhenium(I) triflate
SMILES in allcod.smi: c1cccc2c3cccc[n]3[Re](C#[O])(C#[O])(C#[O])([n]12)[n]1cccc(c1)C(=O)NC1CCCCC1.C(S(=O)(=O)[O-])(F)(F)F

8000096
Name in CIF: anthryldiphosphene
SMILES obtained by OPSIN: P=Pc1c2ccccc2cc2c1cccc2
Correct name: P-(antrhyl)-P'-(2,4,6-tris(bis(trimethylsilyl)methyl)-phenyl)-diphosphene.
SMILES in allcod.smi: P(=Pc1c(cc(cc1C([Si](C)(C)C)[Si](C)(C)C)C([Si](C)(C)C)[Si](C)(C)C)C([Si](C)(C)C)[Si](C)(C)C)c1c2ccccc2cc2ccccc12
